# Limb-specific blood flow regulation during cycling exercise in traumatic single lower limb amputees

**DOI:** 10.1007/s00421-025-05715-3

**Published:** 2025-02-19

**Authors:** Anna Pedrinolla, Fabio Giuseppe Laginestra, Camilla Martignon, Valentina Cavedon, Elisa Fioraso, Caterina Biasiolo, Chiara Milanese, Federico Schena

**Affiliations:** 1 Department of Cellular, Computational, and Integrative Biology – CIBIO., University of Trento, Via Calepina 14, 38122 Trento, Italy; 2 Department of Anesthesiology, University of Utah, Salt Lake City, Utah USA; 3 Department of Neuroscience, Biomedicine, and Movement Sciences, University of Verona, Verona, Italy; 4 Department of Diagnostics and Public Health, University of Verona, Verona, Italy

**Keywords:** Lower-limb amputees, Hemodynamics, Cycling

## Abstract

**Purpose:**

To investigate the limb-specific blood flow regulation during dynamic bilateral cycling exercise in individuals with traumatic single lower-limb amputation compared to a control group.

**Methods:**

Seven individuals with single lower leg amputation (AMP) (37 ± 11 years, 11 ± 8 years since amputation) and 7 age-matched controls (Ctrl) (36 ± 10 years) were tested during three 5 min constant workload exercise at 60W, 100W, and 80% of peak power output (PPO), on a reclined cycling ergometer. In AMP, femoral blood flow (FBF) and leg vascular conductance (LVC) were measured in the amputated leg (AL) and whole leg (WL), while in Ctrl, the same measurements were obtained in the right and left legs. Interlimb balance was measured with a power meter, and bilateral asymmetry index was calculated for FBF and interlimb balance. Oxygen consumption ($\dot{V}$ O_2_), ventilation (${\dot{V}}_{E}$), mean arterial pressure (MAP), heart rate (HR), and cardiac output (CO) were also quantified.

**Results:**

AMP exhibited lower FBF in AL compared to WL (60W, − 61%; 100W, -69%; 80% PPO, − 64%; *p* < 0.001). LVC increased as expected in WL but did not increase significantly throughout workloads in AL. Interlimb balance exhibited a much higher contribution of the WL (60W, 76% of the work; 100W, 68%; 80% PPO,65%) than AL (60W, 26%; 100W, 34%; 80% PPO, 35%). No differences were found in FBF (*p* = 0.187), LVC (*p* = 0.871), and interlimb balance (*p* = 0.829) in CTRLs. No difference between AMP and CTRL in $V$ O_2_ (*p* = 0.241), ${\dot{V}}_{E}$ (*p* = 0.124), MAP (*p* = 0.186), HR (*p* = 0.360), and CO (*p* = 0.144) at any workload was detected.

**Conclusion:**

Individuals with amputation present considerable limb-specific blood flow regulation during bilateral cycling exercise. Understanding the mechanisms for this interlimb difference may provide important information to improve rehabilitation and training in this population.

## Introduction

Being able to respond to increased energy demand during exercise requires an efficient regulation of cardiovascular adjustment and the control of blood flow (Kinzer and Convertino 1989). Indeed, dynamic exercise constitutes a major hemodynamic stress that is met with highly coordinated cardiovascular adjustments to ensure suitable oxygen delivery to contracting skeletal muscles (Hearon and Dinenno 2016). The regulation of blood flow to exercising skeletal muscles depends on the function of the resistance vasculature and a rapid vascular adjustment is needed to increase blood flow to active muscles in which oxygen demand is high, and a concurrent opposite vascular response where oxygen demand is lower, thereby efficiently distributing the proper amount of blood where is needed (Muller-Delp 2016). Thus, due to this fascinating ability to adapt, human beings present a heterogenous blood flow distribution by its nature (Heinonen et al. 2012).

Nowadays, many individuals with traumatic lower-limb amputation are recreationally active or regularly engaged in exercise training and sports, hitting top level categories, and some of them compete in paralympic games (Kars et al. 2009). Recent studies have shown that amputees encounter critical hemodynamic changes (Magalhães et al. 2011; Naschitz and Lenger 2008; Pedrinolla et al. 2023). These alterations are mostly the result of perturbed arterial flow proximal to the amputation site (i.e., change in shear stress, circumferential strain, reflected waves, arterial remodeling, atherosclerosis) interplayed with distal and systemic hemodynamic consequences (i.e., high arterial stiffness, systolic hypertension, increased pulse pressure) (Magalhães et al. 2011; Naschitz and Lenger 2008; Pedrinolla et al. 2023). Indeed, these individuals are typically characterized by dysregulated hemodynamics (Naschitz and Lenger 2008), but also higher arterial stiffness (Magalhães et al. 2011), and last but not least, an important change in vascular structure related to the amount of muscle mass lost with the amputation (Pedrinolla et al. 2023). However, most of the studies that have investigated this population have focused on peak metabolic responses and exercise capacity (Chin et al. 2002; Fischer et al. 2022), walking velocity using different prostheses (Graham et al. 2008; Wezenberg et al. 2019), and mostly metabolic responses during submaximal exercise using a single-leg model (Chin et al. 2002; Wezenberg et al. 2012). Nevertheless, it is not given that individuals with lower limb amputation perform exclusively single-leg exercises (Darter et al. 2013; Elmer and Martin 2021; Fischer et al. 2022). Surprisingly, considering the aforementioned premises, only one study investigated the hemodynamic response to cycling exercise in people with traumatic lower-limb amputation (Storey, Geschwindt, and Astorino 2024), showing that people with a transtibial amputation present similar metabolic and central hemodynamic responses compared to controls. Importantly, to the best of our knowledge, no study has ever assessed the peripheral hemodynamic response to bilateral exercise in people with a unilateral transtibial amputation. Given the tight relationship between limb blood flow and the development of fatigue during exercise (Amann and Calbet 2008), it would be important to quantify whether impairments exist in this population, and whether the amputated limb presents a differential regulation compared to the whole limb. Accordingly, potential interlimb asymmetries (defined as a different response between limbs for a given variable), during bilateral exercise in this population, have never been quantified.

Therefore, the aim of the present study was to compare hemodynamic responses to dynamic bilateral cycling exercise between individuals with single lower-limb amputation and nonamputees, accounted for potential interlimb asymmetry. Our hypothesis was that at the same mechanical and metabolic exercise intensities, individuals with amputation would exhibit lower blood flow responses in the amputated limb than in the whole limb, and that this difference would be maintained when normalized by limb mass. Additionally, this difference would be more pronounced than the physiological between-limbs difference observed in control subjects.

## Methods

### Participants

Individuals were included in the study if subjected to a traumatic lower limb (transtibial) amputation and were free from any other related or unrelated comorbidity. People were also included in the study in the absence of pharmacological therapy, smoking history, atherosclerotic vascular disease, heart failure, and liver, renal, or inflammatory and metabolic diseases. A group of age and sex matched nonamputees was also recruited (CTRL). Exclusion criteria were the same as for the individuals with amputation (AMP). All experiments were conducted after informed and written consent was obtained from the subjects in accordance with the Declaration of Helsinki, as part of a protocol approved by the Institutional Review Board of the Department of Neurosciences, Biomedicine, and Movement Sciences, University of Verona, Italy (Verona, Italy—#CT 27111).

### Study overview

All assessment procedures were performed in the morning between 8.00 am and 12.00 pm, or in the afternoon between 2.00 and 6.00 pm. Subjects visited the lab twice with at least 7 days in between. In the second visit, subjects were asked to arrive at the lab at the same time of the previous visit. For the first time, upon arrival at the Human Anatomy and Exercise Physiology laboratories, subjects were measured for body mass and stature, and underwent body composition assessment using a dual-energy X-ray absorptiometry (DXA). Following the DXA, subjects were asked to sit comfortably on a reclined cycling ergometer (Ergoselect 1200, Ergoline GmbH, Bitz, Germany) and after baseline measurement and a short warm-up, the incremental test to exhaustion was performed to measure exercise capacity. In the second visit, on the same reclined ergometer, individuals performed three 5 min steady-state exercises. During this test, cardiorespiratory variables, hemodynamic blood flow at both common femoral arteries was measured. This ergometer has been selected to allow subjects to perform a bilateral, dynamic, aerobic exercise as well as to allow the measurement of blood flow at the common femoral arteries during steady-state exercise.

### Body composition and limb muscle mass

Fat-free soft tissue mass (FFSTM) was assessed by means of DXA using a total body scanner (QDR Horizon, Hologic MA, USA; fan-beam technology, software for Windows XP version 13.6.). Methods are described somewhere else (Pedrinolla et al. 2023). Briefly, all subjects were required not to undertake strenuous physical activity the day before the measurement session as well as any exercise prior the measurements. Body weight was assessed with the prosthesis to the nearest 0.1 kg using a certified electronic scale (Tanita electronic scale BWB-800 MA, Wunder SA.BI. Srl, Milano, Italy). The weight of the prosthesis was then taken and subtracted from the previous weight with the prosthesis to get the actual body weight. Standing height was measured to the nearest 0.1 cm using a Harpenden portable stadiometer (Holtain Ltd., Crymych, Pembs. UK) according to conventional criteria and measuring procedures (Lohman et al. 1992). Prior to scanning, subjects removed their prostheses, positioning aids to support the residual lower limb were employed, and special strapping was applied around the subjects’ residual ankle to ensure no movement during the scans. Analysis of DXA scans was performed according to the manufacturer’s procedures to get the whole-body FFSTM (expressed in grams) (Pedrinolla et al. 2023).

### Incremental test to exhaustion

To assess exercise capacity, an incremental step test to exhaustion was performed. Once in the lab, subjects were asked to sit on a reclined cycling ergometer (Ergoselect 1200, Ergoline GmbH, Bitz, Germany). AMP performed the test exercising with both legs, using their prosthesis. After two minutes of baseline measurements, a 3–5 min warmup at 50 W was performed. Right after the warmup, the incremental step test started. During the test, the intensity was increased each minute by 20 W until exhaustion. Subjects were asked to keep a cadence between 70 and 80 rpm. The same protocol was used for AMP and CTRL. During the incremental step test, pulmonary gas exchange and ventilatory parameters were measured continuously by means of a metabolic cart (Quark CPET, Cosmed srl, Rome, Italy). Cardiac output (CO) and heart rate (HR) were continuously measured by means of Physioflow (Manatec Biomedical, Poissy, France). Exhaustion was reached in the presence of at least one of the following situations: subject asked to stop, inability to keep pedaling at the given cadence for at least 10 consecutive seconds (below 70 rpm), achievement of a plateau in $\dot{V}$ O_2_ despite an increase in work rate, 95% of the predicted HR_max_, or RER ≥ 1.15 (Edvardsen, Hem, and Anderssen 2014). Peak power output (PPO) was identified as the power corresponding to the last completed step of the incremental step test.

### Constant workload exercise

To assess response to exercise, both AMP and CTRL were asked to perform three 5 min cycling bouts at 60W, 100W, and 80% of peak power output (PPO). This duration was chosen because 5 min is a duration that typically allows obtaining steady-state responses (at low work rates). Also, using both absolute and relative intensities allows more thorough characterization of the exercise responses in this population. Indeed, some of the variables of interest are dictated by absolute intensities (e.g., blood flow, $\dot{V}$ O_2_) while others by relative intensities (e.g., blood pressure, heart rate) (Amann et al. 2014; Smith et al. 2022). All subjects were asked not to undertake strenuous physical activity the day before this assessment. The test was performed at the same reclining ergometer and started with 3 min baseline recording followed by the 3 5 min bouts. The bouts order was counterbalanced to control for sequence effects. During the test, pulmonary gas exchange and ventilatory parameters were measured continuously by means of the same metabolic cart used for the incremental test. Heart rate (HR) and cardiac output (CO) were measured continuously by means of Physioflow (Manatec Biomedical, Poissy, France). For the Physioflow, after gentle skin scraping and as recommended by the manufacturer's instructions, six electrodes were used: 2 on the neck, 2 at the xiphisternum, and one of each side of the chest. HR determination is based on the R–R interval duration determined using the first derivative of the electrocardiogram. Adequate signal quality for interpretation was detected by a color graph. Artifact detection was diagnosed by the Physioflow device and later by one of the investigators who performed manual review of the data and identified data thought to be “physiologically implausible” (i.e., when measuring, for a give subject, values greater than the mean ± 20%) were deleted from the series. An initial calibration procedure of a 30-beat cycle ensured resting HR and CO. The Physioflow software program requires the recording of information: sex, age, height, weight, and systolic and diastolic blood pressure (Brunner-La Rocca 2010; Storey, Geschwindt, and Astorino 2024).

Arterial blood pressure was measured manually at rest and within the last minute of each bout by means of a sphygmomanometer (GIMA, Gima S.p.s., Milano, Italy). Mean arterial pressure (MAP) was calculated using systolic and diastolic blood pressure with the following formula:

MAP (mmHg) = Diastolic blood pressure + ((Systolic blood pressure− Diastolic blood pressure)/3) (Pedrinolla et al. 2023).

Furthermore, at rest and within the last two minutes of each bout, measurements of blood flow at the common femoral artery of both limbs (whole leg and amputated leg in AMP, and right and left in CTRL) were taken using an ultrasound Doppler (GE Logiq-7, General Electric Medical Systems, Milwaukee, WI, USA). Measurements were taken simultaneously by 2 skilled sonographers with a great expertise in ultrasound measurements during exercise, 2 cm above common femoral artery bifurcation (Pedrinolla et al. 2023). Femoral blood flow (FBF) was calculated using arterial diameter and blood velocity according to this formula:

$$FBF \, (ml \cdot min^{ - 1} ) \, = \, V_{mean} \cdot \left( {vessel \, diameter/2} \right)^{2} \cdot 60$$

Blood flow was also normalized for the lower limb muscle mass obtained from DXA (FBF/kg).

Also, to measure pedaling characteristics such as the interlimb balance, a power meter (Vector 3, Garmin Ltd., Kansas, USA) was installed on the reclined cycling ergometer. With these values, it was possible to calculate specific power per limb and further normalize specific limb blood flow (FBF/kg/W).

Leg vascular conductance (LVC) was then calculated with the following formula:

$LVC \, (ml \cdot min^{ - 1} \cdot mmHg^{ - 1} ) \, = \, FBF/MAP$ (Pedrinolla et al. 2023).

*Bilateral asymmetry index*. The bilateral asymmetry index reflects the degree to which the response of one limb differs from the other, indicating that the limb responses may not be equal. This index was calculated for FBF, normalized FBF and right/left limb balance, at any workloads, according to the following formula (Boccia et al. 2022):

$$In \, AMP: \, \left[ {\left( {Whole \, limb \, {-} \, Amputated \, limb} \right)/\left( {Whole \, limb \, + \, Amputated \, limb} \right)} \right] \cdot 100$$

$$In \, CTRL: \, \left[ {\left( {Right \, limb \, {-} \, Left \, limb} \right)/\left( {Right \, limb \, + \, Left \, limb} \right)} \right] \cdot 100$$

Values were considered symmetric when the interlimb difference was < 10%, otherwise they were considered asymmetric, favoring either right/whole limb (WL) or the left/amputated limb (AL) (Boccia et al. 2022).

### Statistical analysis

Data are expressed as mean ± SD if not differently stated. For individuals’ characteristics, unpaired t tests were used to identify between-groups differences (i.e.: anthropometry, body composition, resting arterial blood pressure, maximal exercise capacity and maximal aerobic capacity). A two-way (2 × 3) analysis of variance (ANOVA), with “Group” as between-group factor (AMP and CTRL), and “Intensity” (60W, 100W, and 80%) as within-group factor was used to assess differences in gas exchange and ventilatory parameters, central hemodynamics and MAP. A two-way “4 × 3” ANOVA, with “Limb” (WL, AL, right, and left) as between-group factor, and “Intensity” as within-group factor (60W, 100W, and 80%) was used to assess differences in FBF, LVC and balance between legs.

In the presence of significant effects, multiple comparisons tests with Tukey’s correction were performed. The familywise alpha level for significance was set at 0.05 (two-tails), with Tukey’s correction when needed, for all the analyses. All statistical analyses were performed with GraphPad Prism Version 8.4.3 (Graphpad software LLC., Boston, USA). In the presence of between-limbs differences, Cohen’s d value was calculated to investigate the effect size of the differences in the main outcome (FBF), small (*d* = 0.2), medium (*d* = 0.5), or large (*d* = 0.8).

## Results

### Participants’ characteristics and maximal exercise capacity

Seven single lower leg AMP (37 ± 11 years) and 7 age-matched CTRL (35 ± 9 years) were included in the study. AMP presented a traumatic transtibial amputation (11 ± 8 years since amputation, *n* = 3 right side, *n* = 4 left side, range of time since amputation: minimum 4 years, maximum 19 years) and were free from any medication. None of them suffered from the phantom limb syndrome. Table 1 shows the characteristics of AMP and CTRL. Subjects of both groups were declared to be regularly active and involved in exercise training at least three times a week (i.e.: soccer, running, cycling, rowing, hiking and trail running, mountaineering, and climbing). AMP and CTRL were similar for anthropometry (total weight, height, BMI) and general body composition (whole-body fat and fat-free mass, %) (Table 2). When focusing on the lower limbs, AL showed significant differences compared to WL as well as the control limbs (Table 2). Concerning the incremental test, all the subjects reached their VO_2_max according to the previously cited criteria. Between-groups difference was found for $\dot{V}$ O_2max_ (*p* = 0.041), but this difference disappeared once $\dot{V}$ O_2max_ was normalized for body mass (*p* = 0.179). No between-groups differences in HR_max_ (*p* = 0.614) and CO_max_ (*p* = 0.143) were detected. Additionally, the AMP group reached significantly lower peak power output (PPO) compared to the CTRL group (*p* < 0.001), and this difference persisted after normalizing PPO_max_ for body mass (Table 1).

**Table 1:** Subjects’ characteristics

	AMP	CTRL	
*n*	7	7	*p*-value
Age—years	38 ± 11	35 ± 9	0.581
Dominance—Right/Left		7/0	
Weight- kg	76.9 ± 10.9	82.6 ± 13.2	0.228
Height—cm	177 ± 8	180 ± 4	0.355
BMI—kg∙m^−2^	23.3 ± 3.2	25.2 ± 3.3	0.287
Whole Body Fat Mass—kg	15.1 ± 3.2	14.2 ± 6.0	0.622
Whole Body Free Fat Mass—kg	58.1 ± 8.8	63.9 ± 9.3	0.235
Whole Body Fat Mass—%	19.9 ± 2.9	17.3 ± 4.7	0.728
Whole Body Free Fat Mass—%	76.5 ± 4.5	77.1 ± 5.7	0.747
Rest systolic BP—mmHg	121 ± 6	125 ± 9	0.403
Rest diastolic BP—mmHg	79 ± 6	80 ± 5	0.686
Rest mean arterial BP—mmHg	100 ± 10	104 ± 4	0.156
VO_2max_—L∙min^−1^	2.5 ± 0.5	3.3 ± 0.5	0.041*
VO_2max_—mL⋅min^−1^∙kg^−1^	32.8 ± 5.7	38.1 ± 6.8	0.179
HR_max_—bpm	174 ± 8	175 ± 13	0.614
CO_max_—L∙min^−1^	21.3 ± 4.8	26.1 ± 7.4	0.143
PPO—W	187 ± 37	259 ± 18	< 0.001*
PPO—W∙kg^−1^	2.4 ± 0.5	3.1 ± 0.3	0.041*
80% PPO—W	152 ± 30	209 ± 17	< 0.001*

*BMI* body mass index, *VO*_*2max*_ maximal aerobic capacity, *HR* heart rate, *CO* cardiac output, *PPO* peak power output

^*^Significant between-groups difference

**Table 2:** Anthopometric characteristics

	AMP			CTRL			
	AL	WL	Within *p*-value	Right	Left	Within p-value	Between *p*-value
Mass Tot—kg	8.3 ± 1.9	12.9 ± 2.2	< 0.001 *	13.7 ± 1.9	13.3 ± 1.9	0.783	< 0.001 *
Fat Mass—kg	2.1 ± 0.5	2.5 ± 0.7	0.127	2.7 ± 1.1	2.6 ± 1.1	0.832	0.002 *
Free Fat Mass—kg	6.0 ± 1.5	9.8 ± 1.9	< 0.001 *	10.5 ± 0.9	10.1 ± 1.1	0.796	0.002 *
Fat Mass—%	25 ± 5	19 ± 4	0.127	19 ± 6	19 ± 6	0.832	0.134
Free Fat Mass—%	72 ± 4	76 ± 5	0.673	76 ± 6	76 ± 6	0.796	0.239

Between group differences were noted only between AL and both CTRl limbs (Right and Left). For this reason, between p-values refer to the difference between AL and average of Right and Left limbs of Ctrl group

*AL* amputated leg, *WL* whole leg

^*^Significant between AL and Ctrl limbs

### Interlimb balance

**Fig. 1:**
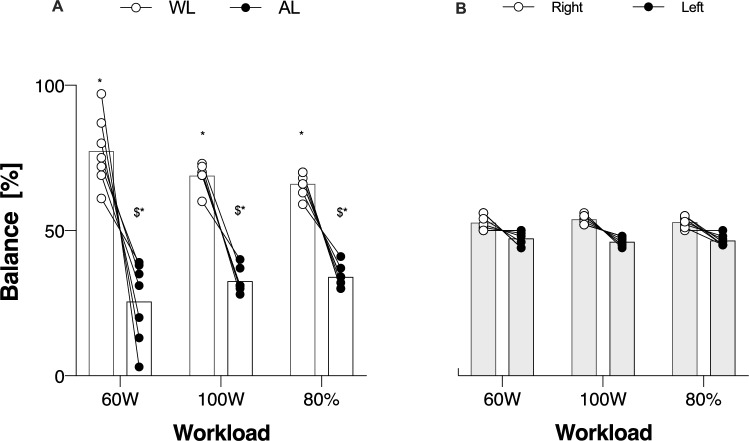
Interlimb balance between Amputated (AL) and Whole Limb (WL) and Right and Left limbs in Amp (panel A) and Ctrl (Panel B). $: significantly different from WL; *significantly different from CTRL

Main effects for interlimb balance were found for “Limb” (*p* < 0.001, *F* = 90.9), but not for “Intensity” (*p* = 0.790, *F* = 0.2). There was a significant interaction between “Limb” and “Intensity” (*p* = 0.001, *F* = 56.8). AMP group involved more WL compared to AL at any workload (60W- Mean Diff: 51.7, *p* < 0.001, *d* = 1.8; 100W- Mean Diff: 36.3, *p* = 0.002, *d* = 1.9; 80%—Mean Diff: 32.0, *p* = 0.001, *d* = 1.9) (Fig. 1, panel A). Also, AL exhibited higher involvement at 100W compared to 60W (Mean Diff: 7.0, *p* = 0.022), and at 80% compared to 60W (Mean Diff: 8.4, *p* = 0.004). CTRL exhibited a quite balanced riding involving right and left limbs almost equally, with no differences between limbs (Fig. 1, panel B).

### Femoral blood flow during submaximal constant workload exercise

**Fig. 2:**
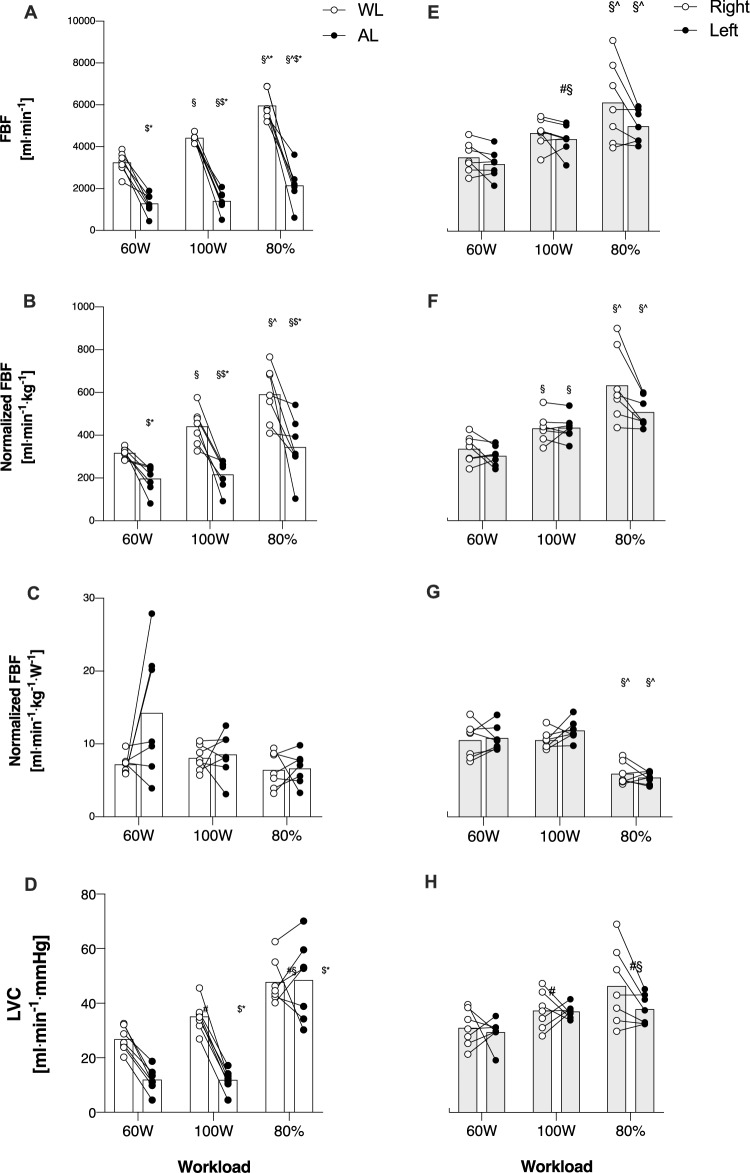
Femoral artery blood flow (FBF), also normalized for muscle mass, as well as for Watts in Amputated (AL), Whole leg (WL), Right, and Left leg respectively in Amp (panels A, B, and C) and Ctrl (panels D, E, and F). §: significantly different from 60W; ^ = significantly different form 100W; $: significantly different from WL; *significantly different from CTRL

Main effects for FBF were found for “Intensity” (*p* < 0.001, *F* = 268.9), and “Limb” (*p* < 0.001, *F* = 34.5). There was a significant interaction between “Intensity” and “Limb” (*p* < 0.001, *F* = 911.87). Significant differences in FBF were found between 100 and 60W (Mean Diff: 900.2, *p* < 0.001), 80% and 60W (Mean Diff: 1983.0, *p* < 0.001), as well as 80% and 100W (Mean Diff: 1082.1, *p* = 0.001). FBF was significantly lower in AL compared to WL, at any workloads (60W, Mean Diff: − 1963.1, *p* < 0.001, *d* = 1.8; 100W, Mean Diff: −3016.2, *p* < 0.001, *d* = 1.9; 80%, Mean Diff: 3815.1, *p* < 0.001, *d* = 1.8). (Fig. 2, panel A). Also, AL exhibited significantly lower values compared to both right and left limbs at any workload (60W, Mean Diff: −876.3, *p* = 0.002; 100W, Mean Diff: 2001.3, *p* = < 0.001; 80%, Mean Diff: 1403.1, *p* = 0.004). No difference between WL and ctrl limbs was detected. No differences between right and left limbs were observed in the CTRL group at any workloads (Fig. 1, panel E).

Main effects for FBF/kg were found for “Intensity” (*p* < 0.001, *F* = 276.4) and “Limb” (*p* < 0.001, *F* = 10.45). There was a significant interaction between “Intensity” and “Limb” (*p* < 0.001, *F* = 6.19). Significant differences in FBF/kg were found between 100 and 60W (Mean Diff: 91.6, *p* < 0.001), 80% and 60W (Mean Diff: 228.3, *p* < 0.001), as well as 80% and 100W (Mean Diff: 136.7, *p* < 0.001). FBF/kg was significantly lower in AL compared to WL at any workloads (60W, Mean Diff: − 120.1, *p* < 0.001, *d* = 1.6; 100W, Mean Diff: − 225.4, *p* < 0.001, *d* = 1.6; 80%, Mean Diff: − 247.2, *p* < 0.001, *d* = 1.4) (Fig. 2, panel B). Also, AL exhibited significantly lower values compared to both right and left limbs at any workload (60W, Mean Diff: −120.5, p = 0.028; 100W, Mean Diff: 208.6, *p* = 0.003; 80%, Mean Diff: − 200.4, *p* = 0.025). No differences between right and left limbs were observed in the CTRL group at any workloads (Fig. 1, panel F).

Main effects for FBF/kg/W were found for “Intensity” (*p* < 0.001, *F* = 30.87), but not for “Limb” (*p* = 0.297, *F* = 1.3). No interactions between “Intensity” and “Limb” were found (*p* = 0.302, *F* = 1.4). Significant differences were found only between 80% and 60W (Mean Diff: 4.53, *p* < 0.001), and between 80% and 100W (Mean Diff: 3.58, *p* < 0.001). No between-limbs differences were detected in any group at any workload (Fig. 2, panel C and G).

### Metabolic and ventilatory response during submaximal constant workload exercise

Main effects for $\dot{V}$ O_2_ were found for “Intensity” (*p* < 0.001, *F* = 203.8) but not for “Group” (*p* = 0.233, *F* = 1.6). The same results were found for $\dot{V}$ CO_2_, where the main effect was found for “Intensity” (*p* < 0.001, *F* = 206.6) but not for “Group” (*p* = 0.275, *F* = 1.3). Main effects for ${\dot{V}}_{E}$ were found for “Intensity” (*p* < 0.001, *F* = 20.9) but not for “Group” (*p* = 0.527, *F* = 0.4). In both groups, $\dot{V}$ O_2_, $\dot{V}$ CO_2_, and ${\dot{V}}_{E}$ at 100W were higher than 60W (*p* < 0.001), as well as 80% were higher than 60W (*p* < 0.001) and 100W (*p* < 0.001) (Table 3).

**Table 3:** Oxygen consumption, ventilation, central hemodynamics, and mean arterial pressure at rest and during exercise in AMP and CTRL

	VO_2_—ml⋅min^−1^			VE—L⋅min^−1^			HR—bpm			CO—L⋅min^−1^			MAP—mmHg		
	AMP	CTRL	*p*-value	AMP	CTRL	p-value	AMP	CTRL	*p*-value	AMP	CTRL	*p*-value	AMP	CTRL	*p*-value
60W	1227 ± 49	1225 ± 165	0.751	32.1 ± 5.3	32.8 ± 4.8	0.751	87 ± 24	91 ± 13	0.851	7.9 ± 1.8	10,3 ± 2.9	0.144	106 ± 9	111 ± 5	0.548
10W	1763 ± 106	1639 ± 170	0.241	52.2 ± 9.7	45.8 ± 5.3	0.124	124 ± 15	109 ± 15	0.360	11.9 ± 2.1	13.7 ± 1.2	0.149	115 ± 9	116 ± 5	0.957
80%	2396 ± 556	2610 ± 457	0.455	77.6 ± 17.5	86.1 ± 8.2	0.544	167 ± 13	167 ± 10	0.887	16.7 ± 2.8	18.5 ± 2.0	0.129	121 ± 10	120 ± 9	0.763

### Central hemodynamic response during submaximal constant workload exercise

Main effects for HR were found for “Intensity” (*p* < 0.001, *F* = 238.8), but not for “Group” (*p* = 0.636, *F* = 0.2). Main effects for CO were found for “Intensity” (*p* < 0.001, *F* = 182.5), and for “Group” (*p* = 0.017, *F* = 7.23). For both HR and CO, no interaction between “Intensity” and "Group” was found. In both groups, HR and CO at 100W were higher than 60W (*p* < 0.001), as well as 80% were higher than 60W (*p* < 0.001) and 100W (*p* < 0.001) (Table 3).

### Mean arterial pressure during submaximal constant workload exercise

Main effect for MAP was found for “Intensity” (*p* = 0.038, *F* = 3.1) but not for “Group” (*p* = 0.763, *F* = 0.4). In both groups, MAP was higher only at 80% compared to 60W (*p* = 0.026) (Table 3).

### Leg vascular conductance during submaximal constant workload exercise

Main effects were found for “Intensity” (*p* < 0.001, *F* = 73.81) and “Group” (*p* = 0.003, *F* = 9.28). Interactions between “Intensity” and “Group” were also found (*p* < 0.001, *F* = 10.73). Significant differences were found between 100 and 60W (Mean Diff: 5.58, *p* < 0.001), 80% and 60W (Mean Diff: 20.4, *p* < 0.001), as well as 80% and 100W (Mean Diff: 14.8, *p* = 0.002). LVC was significantly lower in AL compared to WL at 60W (Mean Diff: − 14.9, *p* = 0.002) and at 100W (Mean Diff: 23.2, *p* = 0.002). No differences were detected at 80% between AL and WL (Fig. 2, panel D). LVC was significantly lower in Al compared to both ctrl limbs at 60W (Mean Diff: − 18.5, *p* < 0.001) and at 100W (Mean Diff: − 25.8, *p* < 0.001). No differences were detected at 80% between AL and both limbs of the CTRL group. CTRL did not exhibit any difference between limbs (right vs left) (Fig. 2, panel H).

### Bilateral asymmetry index

Bilateral asymmetry index was calculated in both groups for right/left and WL/AL. AMP exhibited great asymmetry index at any workloads compared to CTRL. Indeed, FBF in AMP shows an asymmetry in favor of WL (60W = 46 ± 17%; 100W = 53 ± 13%; 80% = 48.8 ± 17.1%; *p* < 0.0001). FBF/kg also exhibited great asymmetry index at any workload (60W = 24 ± 17%; 100W = 32 ± 17%; 80% = 26.3 ± 22%; *p* < 0.001). Great asymmetry index was found also in interlimb balance, but it decreased at the highest workload (60W = 49 ± 24%; 100W = 34 ± 9%;80% = 29.0 ± 11.2; *p* < 0.0001).

Conversely, CTRL did not exhibit any asymmetry between limbs in FBF, FBF/kg, or interlimb balance. Indeed, asymmetry indexes calculated for these parameters at 60W, 100W, and 80% were negligible, being always lower than 10%. Furthermore, no differences between 60W, 100W, and 80% were detected in CTRL.

## Discussion

Although other studies have examined the central hemodynamic response in individuals with lower-limb amputation (Storey, Geschwindt, and Astorino 2024), to our knowledge, this is the first study to investigate blood flow distribution during dynamic bilateral cycling exercise in individuals with unilateral traumatic lower-limb amputation (AMP) compared to controls (CTRL), with a focus on potential interlimb differences. In the present study, AMP and CTRL were compared for three 5 min bouts at 60W, 100W, and 80% of PPO while measuring blood flow at both common femoral arteries. Metabolic, ventilatory, and central and peripheral hemodynamic responses were also measured. Interlimb balance was measured observing the contribution of each limb while exercising on the reclined cycling ergometer. Additionally, blood flow response to exercise was normalized for the power exerted by each limb with the aim of better understanding the hemodynamic response in this population. Bilateral asymmetry index was then calculated for blood flow, blood flow normalized for muscle mass, and interlimb balance. In accordance with our initial hypothesis, AMP exhibited significantly different distribution of blood flow between AL and WL. This happened despite a similar central hemodynamic response and a similar metabolic response compared to CTRL at both absolute and relative exercise intensity (60, 100W, and 80%PPO). Still, blood flow in AL did not increase in the same way it increased in WL and in both limbs of CTRL. This trend was confirmed even when blood flow was normalized for lower limb muscle mass. However, the difference between AL and WL, as well as between AL and both limbs of CTRL, disappeared once blood flow was further normalized for the power exerted by each limb. This normalization resulted in equal blood flow to each limb when accounting for the amount of muscle mass engaged and the watts exerted. This finding represents a novel insight into the hemodynamic response during bilateral cycling exercise in this population. Further, this supports the idea that individuals with lower-limb amputation, when able to maintain an active lifestyle like those included in our study, can efficiently regulate blood flow between limbs in response to specific limb metabolic demand variation.

### Exercise capacity in individuals with traumatic single lower-limb amputations

Results of our study show a 25% reduction in PPO in AMP compared to CTRL. Differences were found concerning maximal aerobic capacity, with AMP showing lower values compared to CTRL. However, this difference disappeared once maximal aerobic capacity was normalized for body mass. These results are partially supported by previous literature. A review by Fischer and colleagues (2022) examined the peak response and the metabolic response during running in people with amputation and controls. A similar $\dot{V}$ O_2max_ despite differences in achieved peak speed was found. Other studies have investigated exercise capacity in people with traumatic lower-limb amputation showing poorer results compared to controls concerning both maximal workload and aerobic capacity, confirming our results. Chin and colleagues (2002) reported a maximal workload reduced by 33% in people with amputation compared to controls, accompanied by $\dot{V}$ O_2max_ reduced by 20%. However, in the study by Chin and colleagues (2002), a one-leg cycling test driven by the whole leg was performed, while controls performed the test using exclusively the right leg. A more recent study by Wezenberg and colleagues (2012) measured PPO and $\dot{V}$ O_2max_ in people with traumatic amputation and a control group, but no difference was detected. Still, exercise capacity was tested during a one-leg exercise test during that people with amputation performed with the whole leg, and controls performed with one leg randomly assigned (Wezenberg et al. 2012).

### Blood flow response to exercise in individuals with traumatic lower-limb amputations

Our results show a different blood flow distribution between limbs in AMP compared to CTRL. Indeed, AMP exhibited more than 60% reduced blood flow in the AL compared with WL (Fig. 2, panel A). This might be the result of a significantly reduced muscle mass to irrorate with blood and nutrients (Pedrinolla et al. 2023). However, the difference between AL and WL persisted even when blood flow was normalized for muscle mass, suggesting that blood flow regulation during exercise might not be just a matter of muscle mass (Fig. 2, panel B). In this study, the interlimb balance was also measured to verify how much each limb was involved in the exercise. In CTRL, blood flow was uniformly distributed between Right and Left (Fig. 1, panels E and F), and Right and Left limbs were involved almost equally confirmed even by bilateral asymmetry, which was lower than 10%, so negligible. In AMP, WL was much more involved in the exercise than the AL taking the 76% of the work at 60W, the 67% of the work at 100W, and the 65% at 80%PPO (Fig. 1, panel A). Interlimb (un)balance was confirmed by bilateral asymmetry as well, markedly in favor of WL. Interestingly, once blood flow was further normalized for the power exerted by each limb (calculated from data retrieved from the power meter), the previously significant differences between limbs disappeared, showing similar blood flow to each limb for every kilogram of muscle mass engaged and for each watt exerted by the individual limb. These results suggest that the amputated limb does not possess an “intrinsic” impairment to receive blood flow, but on the contrary, it only receives the appropriate amount of blood flow required based on the muscle mass involved and the work that the limb is performing. This finding is entirely novel as no previous studies have explored this specific aspect of hemodynamic response in individuals with lower-limb amputation. As such, our data cannot be directly compared to existing results in the literature.

### Metabolic, ventilatory, and central hemodynamic response to exercise in individuals with lower-limb amputation

Results of this study did not show any difference in metabolic and central hemodynamic response during bipodal cycling on a reclined ergometer at 60W, 100W, and 80% PPO between AMP and CTRL. Even though CO appeared to be numerically greater in CTRL, this difference was not statistically significant, which could be related to our study likely being underpowered for these secondary outcomes. Indeed, a lower CO response in AMP at the same work rate could potentially be justified by the lower blood flow directed to the amputated limb, and likely not stemming from an impaired response. This further highlights the importance of studying peripheral blood flow in conjunction with central hemodynamics.

Our results are supported by previous studies, even if several different ergometers or exercise modalities were used. Kinzer and Convertino (1989), compared people with amputation and controls during a 35W steady-state exercise on an arm-crank ergometer. They measured VO_2_ and CO and the no between-groups differences were found (Kinzer and Convertino 1989). Also, Kurdibaylo (1994) measured cardiorespiratory response in people with amputation and controls during submaximal exercise on a wheelchair ergometer showing similar HR and VE response at all submaximal intensities. More recently, Esposito and colleagues (2014) measured VO_2_ and HR during five walking velocity conditions in people with transtibial amputation and a control group, reporting no between-groups differences. Only one case study, by Elmer and Martin (2021), measured metabolic power and efficiency during submaximal single-leg cycling with counterweight in an individual with transpelvic amputation. The subject performed 4 steady-state bouts, including one at 100W at which he exhibited the same VO_2_ of the individuals included in our study (Elmer and Martin 2021).

### Mechanisms of blood flow regulation during exercise in individuals with lower-limb amputation

Studies on cardiovascular response to exercise explain very well how blood flow is usually not uniformly distributed within the skeletal muscles during dynamic exercise in humans (Heinonen et al. 2012; Laughlin 1999; Muller-Delp 2016). Certainly, blood flow heterogeneity is a functionally essential element that aims to efficiently deliver oxygen and nutrients to the tissues (Heinonen et al. 2012). Regulation of skeletal muscle blood flow and thus oxygen delivery to contracting muscles is complex and involves several finely tuned mechanisms including vascular regulation, mechanical effects of muscle contraction, local metabolic changes, and sympathetic nervous activity (Heinonen et al. 2012; Laughlin 1999). Considering vascular mechanisms, a decreased vascular resistance to flow through the vascular tree, is the primary cause of increase in blood flow to active muscle, which is reflected by an important increase in vascular conductance, mostly mediated by relaxation of smooth muscles in resistance arteries and arterioles feeding the active skeletal muscles (Laughlin 1999). Additionally, vascular conductance is tightly coupled to metabolic rate so that conductance increases with increased muscle activity and therefore metabolic rate of muscle plays a key role in control of blood flow during exercise (Laughlin 1999).

The results of this study show that in individuals with traumatic lower-limb amputation, blood flow distribution is regulated according to limb involvement during bilateral dynamic cycling exercise, with blood flow matched to the work rate performed by each limb. Indeed, blood flow to the working limbs was apparently distributed in favor of WL rather than AL (Fig. 2, panels A and B). Indeed, blood flow was equally distributed to both AL and WL, based on the amount of work performed by each limb. This might be the results of a finely tuned mechanisms to properly regulate the amount of absolute blood toward a specific limb made of several interplayed events, such as reduced vascular conductance in AL (Fig. 2, panel D), synonymous of elevated vascular resistance, and reduced muscular activity as supported by the less involvement of the AL during exercise (Fig. 1, panel A) which probably led to a minor request of oxygen from the exercising muscle in the amputated limb and a consequent lower metabolic rate. From this point of view, diminished absolute blood flow to amputated limb does not represent dysfunctional hemodynamics but rather precise physiological response to provide the right amount of blood to each limb.

### Limits of the study

The main limit of this study is probably the inadequate sample size. Indeed, considering the poor literature available on this population, no information to calculate statistical power was retrieved. Also, the selected population was not easy to recruit, making it difficult to include a larger number of subjects. Thus, a convenience sample size was chosen based on the feasibility of the recruitment. Also, it has to be considered that the experimental design was somewhat challenging and only individuals motivated in engaging in exercise volunteered in this project, making these results not applicable to the majority of this population. Furthermore, no women volunteered in this study, making these results generalizable only to men with amputation. Another limit consists of not having recorded perceived general or local (i.e., limb-specific) fatigue. This information might have provided important insights to understand constant load exercise. Lastly, this study does not provide information about oxygen extraction of each limb, which would have helped in understanding better this hemodynamic response.

## Conclusions

To our knowledge, this is the first study to assess limb-specific blood flow response to exercise in people with traumatic single lower-leg amputation compared to controls. Results of this study confirm our hypothesis showing that people with amputation present substantial absolute interlimb differences in peripheral hemodynamics during bilateral cycling exercise. However, the differences disappeared once the blood flow was normalized for the work performed by each limb, showing that blood flow regulation in this population is still efficiently tuned with metabolic demand by working muscles. Nevertheless, further studies designed to investigate the mechanisms beneath the observed responses are needed and would provide precious information to implement rehabilitation procedures and training methods for individuals with single lower limb amputation.

## Data Availability

All data generated or analyzed during this study are included in this published article.
